# Supplementary material for: The Evolution and Characterization of the RNA Interference Pathways in Lophotrochozoa
Source: Genome Biol Evol. 2024 May 7;16(5):evae098. doi: 10.1093/gbe/evae098 (PMC11114477; doi:10.1093/gbe/evae098)
Supplement: evae098_Supplementary_Data [file evae098_supplementary_data.zip › Supplementary_materials.zip!/selected_miRNAs/CrGi_selected_miRNAs/CrGirGi_NC_047559.1_2353.pdf]

Provisional ID	:	NC_047559.1_2353
Score total	:	522.3
Score for star read(s)	:	3.9
Score for read counts	:	515
Score for mfe	:	2.3
Score for randfold	:	1.6
Score for cons. seed	:	-0.6
Total read count	:	1022
Mature read count	:	747
Loop read count	:	0
Star read count	:	275

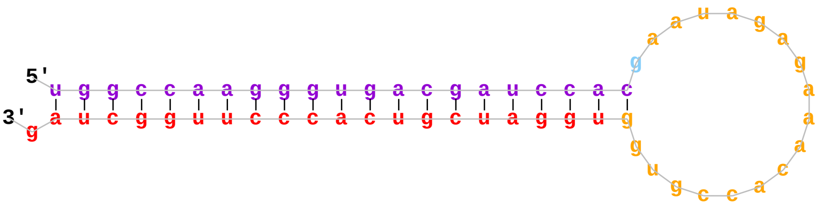

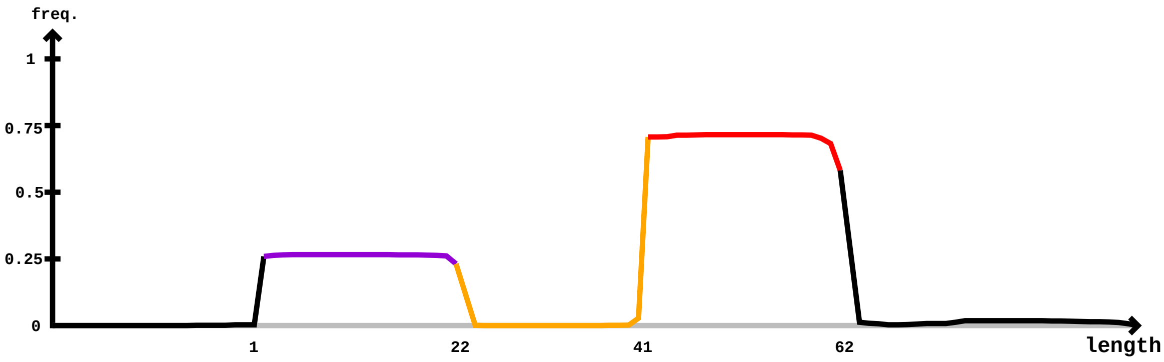

Star Mature

```
5'- gagagagagagaguccucgcuggccaagggugacgauccacgaauagagaaacaccgugguggaucgucacccuuggcuagcgaagagagacagagagagagagagagaga   -3'   obs
    gagagagagagaguccucgcuggccaagggugacgauccacgaauagagaaacaccgugguggaucgucacccuuggcuagcgaagagagacagagagagagagagagaga         exp
    ............((((((((((((((((((((((((((((((....................)))))))))))))))))))))))...))).)))................. reads   mm   sample
    ....................uggccaagggugacgauc.........................................................................  1      0    Ov1
    ....................uggccaagggugacgaucca.......................................................................  11     0    Ov1
    ....................uggccaagggugacgauccac......................................................................  34     0    Ov1
    ....................ugAccaagggugacgauccac......................................................................  1      1    Ov1
    ....................uggccGagggugacgauccac......................................................................  1      1    Ov1
    ....................uggccaagggugacgauccacg.....................................................................  1      0    Ov1
    .....................ggccaagggugacgauccac......................................................................  1      0    Ov1
    ......................gccaagggugacgauccac......................................................................  1      0    Ov1
    ......................gccaagggugacgauccacgC....................................................................  1      1    Ov1
    ...........................................................guggaucgucacccuuggcu................................  2      0    Ov1
    ...........................................................guggaucgucacccuuggcua...............................  5      0    Ov1
    ...........................................................guggaucgucacccuuggcuag..............................  2      0    Ov1
    ...........................................................guggaucAucacccuuggcuagcga...........................  2      1    Ov1
    ............................................................uggaucgucacccuuggc.................................  3      0    Ov1
    ............................................................uggaucgucacccuuggcu................................  4      0    Ov1
    ............................................................uggaucgucacccuuggcua...............................  23     0    Ov1
    ............................................................uggaucgucacccuuggcuag..............................  99     0    Ov1
    ............................................................Cggaucgucacccuuggcuag..............................  1      1    Ov1
    ............................................................Aggaucgucacccuuggcuag..............................  1      1    Ov1
    ............................................................uggaucgucacccuuggcuGg..............................  2      1    Ov1
    ...............................................................aucgucacccuuggcuagcgaa..........................  1      0    Ov1
    .......................................................................................gagaAagagagagagagagaga..  1      1    Ov1
    ............................................................................................agagagagagagagagag.  1      0    Ov1
    .............................................................................................gagagagagagagagaga  3      0    Ov1

    ....................uggccaagggugacgaucca.......................................................................  12     0    Ov2
    ....................Cggccaagggugacgauccac......................................................................  1      1    Ov2
    ....................uggccaGgggugacgauccac......................................................................  1      1    Ov2
    ....................uggccGagggugacgauccac......................................................................  2      1    Ov2
    ....................uggccaaggAugacgauccac......................................................................  1      1    Ov2
    ....................uggccaagggugacgauccGc......................................................................  1      1    Ov2
    ....................uggccaagggugacgauccac......................................................................  44     0    Ov2
    .....................ggccGagggugacgauccac......................................................................  1      1    Ov2
```

Star **Mature**

gagagagagagaguccucgcuggccaagggugacgauccacgaauagagaaacaccgugguggaucgucacccuuggcuagcgaagagagacagagagagagagagagaga			
.....................ggccaagggugacgauccac..	1	0	Ov2
....guggaucgucacccuuggcua...............................	1	0	Ov2
....guggaucAucacccuuggcuag..............................	1	1	Ov2
....guggaucAucacccuuggcuagcga...........................	1	1	Ov2
....guggaucgucacccuuggcuagcgaa..........................	1	0	Ov2
....uggaucgucacccuuggc.................................	2	0	Ov2
....uggaucgucacccuuggcu................................	4	0	Ov2
....uggaucgucacccuuggcua...............................	17	0	Ov2
....uggaucgucacccuuggcuGg..............................	3	1	Ov2
....uggaucguUacccuuggcuag..............................	1	1	Ov2
....uggaucgucGcccuuggcuag..............................	1	1	Ov2
....Aggaucgucacccuuggcuag..............................	1	1	Ov2
....uggaucgucacccuuggcuag..............................	105	0	Ov2
....uggaucgucaccAuuggcuag..............................	1	1	Ov2
....uggaucgucacccuuggcuagc.............................	1	0	Ov2
....Gggaucgucacccuuggcuagc.............................	1	1	Ov2
....uggaucgucacccuuggcuagcU............................	1	1	Ov2
....gaucgucacccuuggcuagc.............................	1	0	Ov2
....aucgucacccuuggcuagcgaa..........................	1	0	Ov2
....cgucacccAuggcuagcgaag.........................	1	1	Ov2
....gucacccuCggcuagcgaa..........................	1	1	Ov2
....agGgagacagagagagag.........	1	1	Ov2
....agagacagagagagagagG......	1	1	Ov2
....agagagagagagagagag.	1	0	Ov2
.................cgcuggccaagggugacgau..	1	0	Mt2
.................cgcuggccaaggUugacgaucca...	1	1	Mt2
....................uggccaagggugaUgaucc..	1	1	Mt2
....................uggccaagggugacgaucca...	4	0	Mt2
....................uggccaagggugacgauccac..	64	0	Mt2
....................Aggccaagggugacgauccac..	1	1	Mt2
....................uggccGagggugacgauccac..	2	1	Mt2
....................uggccaagggugaUgauccac..	2	1	Mt2
.....................ggccGagggugacgauccac..	1	1	Mt2
....gugguggaucgucacccuu....................................	1	0	Mt2
....Aguggaucgucacccuuggcu................................	1	1	Mt2
....guggaucgucacccuuggc.................................	1	0	Mt2
....guggaucAucacccuuggcu................................	1	1	Mt2
....guggaucgucacccuuggcua...............................	2	0	Mt2
....guggaucAucacccuuggcuag..............................	1	1	Mt2
....uggaucgucacccuuggc.................................	5	0	Mt2
....uggaucgucacccuuggcu................................	6	0	Mt2
....uggaucgucacccuuggcua...............................	23	0	Mt2
....uggaucgucacccuuggUuag..............................	1	1	Mt2
....uggCucgucacccuuggcuag..............................	2	1	Mt2
....uAgaucgucacccuuggcuag..............................	1	1	Mt2
....uggaucgucacccuuggcuGg..............................	2	1	Mt2
....uggaucgucacccuuggcuag..............................	181	0	Mt2
....aucgucacccuuggcuagcg............................	1	0	Mt2
....aucgucacccuuggcuagcga...........................	1	0	Mt2
....agagagaAagagagagagag.......	1	1	Mt2
....agacagagagagagagag.....	1	0	Mt2
....gUcagagagagagagagaga..	1	1	Mt2
....agagagagagagagagag.	1	0	Mt2
....gagagagagagagagaga	2	0	Mt2
.............ucUucgcuggccaagggugac...	1	1	Mt1
....................uggccaagggugacgaucc..	1	0	Mt1
....................uggccaagggugacgaucca...	3	0	Mt1
....................uggccaagggugacgauccGc..	2	1	Mt1
....................Aggccaagggugacgauccac..	1	1	Mt1
....................uggccaagggugacgauccac..	72	0	Mt1
....................uggccGagggugacgauccac..	1	1	Mt1
....................uggccaGgggugacgauccac..	2	1	Mt1
....................uggccaagggugacUauccac..	1	1	Mt1
.......................ccaagggugacgauccac..	1	0	Mt1
....guggaucgucacccuugg..................................	1	0	Mt1
....guggaucAucacccuuggcu................................	1	1	Mt1
....guggaucgucacccuuggcu................................	1	0	Mt1
....guggaucgucacccuuggcua...............................	3	0	Mt1

```
                    Star                                        Mature
gagagagagagaguccucgcuggccaagggugacgauccacgaauagagaaacaccgugguggaucgucacccuuggcuagcgaagagagacagagagagagagagagaga
...........................................................guggaucAucacccuuggcuag..............................    1     1     Mt1
............................................................uggaucgucacccuuggc.................................    1     0     Mt1
............................................................uggaucgucacccuuggcu................................    1     0     Mt1
............................................................uggaucgucacccuuggcua...............................    30    0     Mt1
............................................................uggaucgucacccuuggGua...............................    1     1     Mt1
............................................................uggaucUucacccuuggcuag..............................    1     1     Mt1
............................................................uggaucgucacccuuggcuGg..............................    4     1     Mt1
............................................................uggaucgucacccuuggcuag..............................    176   0     Mt1
............................................................uggaucgucacccuuggcuagc.............................    4     0     Mt1
...............................................................aucgucacccuuggcuagcg............................    2     0     Mt1
........................................................................................agacagagagagagagagag...    1     0     Mt1
.........................................................................................gacagagagagagaCagagag.    1     1     Mt1
............................................................................................agagagagagagagagag.    1     0     Mt1
............................................................................................agagagagagagagagaga    1     0     Mt1
.............................................................................................gagagagagagagagagC    1     1     Mt1
```
